# Cerebral microbleeds in traumatic brain injury: their impact on white matter integrity assessed by diffusion MRI

**DOI:** 10.3389/fneur.2025.1630427

**Published:** 2025-09-10

**Authors:** Dávid Bognár, Zalán Petneházy, Péter Laár, Tamás Dóczi, Attila Schwarcz, Bálint S. Környei, Arnold Tóth

**Affiliations:** ^1^Medical School, University of Pécs, Pécs, Hungary; ^2^Department of Medical Imaging, University of Pécs, Pécs, Hungary; ^3^National Laboratory of Translational Neuroscience RRF-2.3.1-21-2022-00011, Pécs, Hungary; ^4^Department of Neurosurgery, University of Pécs, Pécs, Hungary

**Keywords:** traumatic brain injury, traumatic axonal injury, diffusion tensor imaging, cerebral microbleeds, white matter integrity, secondary axotomy

## Abstract

**Introduction:**

Traumatic Brain Injury (TBI) often leads to lasting cognitive and functional deficits, with Traumatic Axonal Injury (TAI) being a significant prognostic factor. This study investigated white matter microstructural changes in moderate-to-severe TBI, focusing on the presence and number of cerebral microbleeds (MBs) using diffusion tensor imaging (DTI).

**Materials and methods:**

51 participants were recruited and categorized into three groups: 17 controls, 17 TBI patients with MBs (MBP), and 17 TBI patients without MBs (MBN). Age matching was applied to minimize confounding effects. MRI scans were acquired using a 3 T Siemens MAGNETOM Prisma scanner, and DTI data were preprocessed using FSL software. Whole white matter and corpus callosum masks were reconstructed using FreeSurfer, while tractography-based methods were implemented with FSL. Fractional anisotropy (FA) and mean diffusivity (MD) were extracted and compared across groups. Group-level voxel-wise statistical analysis was conducted using Tract-Based Spatial Statistics (TBSS), and generalized linear models (GLiMs) were applied to assess the effects of age, sex and MB number on DTI parameters.

**Results:**

Significant decrease in FA (*p* = 0,008 − 0,042) and increases in MD (*p* = 0,004 − 0,016) were observed in the WM masks when comparing the MBP group with the controls. In the TBSS analysis FA (*p* = 0,008) and MD (*p* = 0,005) showed significant differences between the MBP-CON comparison, while FA (*p* = 0,012) and MD (*p* = 0,043) were significantly different between the MBP and MBN groups. Moreover, a significant FA decrease was observed in the corpus callosum when comparing the MBP and MBN groups (*p* = 0,007). Additionally, an increasing number of microbleeds was significantly associated with altered DTI metrics in across all white matter masks.

**Conclusion:**

Our findings highlight MBs as potential markers of more extensive white matter injury in moderate-to-severe TBI. The increase in MBs suggests even greater white matter damage, indicating a progression of microstructural alterations. On a global scale, tractography enhances the sensitivity in detecting structural alterations compared to traditional segmentation techniques. Examination of central white matter areas holds significant importance in uncovering the relevance of MBs.

## 1 Introduction

Traumatic Brain Injury (TBI) remains a significant global health issue, affecting millions of individuals annually and often resulting in long-term cognitive and functional impairments. Among the various white matter (WM) pathologies associated with TBI, Traumatic Axonal Injury (TAI) plays a significant role in determining prognosis (1). Its clinical presentation highly variable, ranging from transient loss of consciousness to severe cognitive dysfunction, depending on the severity of the underlying lesions (2).

Although well-characterized in experimental models, the complex and widespread molecular mechanisms of axonal degeneration, cannot be directly visualized in the clinical setting. Conventional neuroimaging techniques lack the sensitivity to detect the microstructural and biochemical alterations underlying TAI. However, diffusion MRI techniques, including Diffusion Tensor Imaging (DTI), offer a valuable opportunity to indirectly assess these otherwise undetectable alterations. By measuring the directionality and magnitude of water diffusion within WM tracts, DTI enables the identification of microstructural abnormalities linked to cytoskeletal disruption, demyelination, and axonal degeneration. Therefore, DTI represents a valuable tool for translating these intricate molecular processes into clinically relevant biomarkers of WM integrity following TBI. Numerous DTI studies have demonstrated that TBI patients frequently exhibit reduced fractional anisotropy (FA) and increased mean diffusivity (MD), radial diffusivity (RD), and axial diffusivity (AD) across various WM regions. These findings provide further evidence of microstructural damage and highlight the utility of DTI in assessing TAI (3–9).

TAI is often accompanied by microbleeds (MBs), which can be visualized using susceptibility-weighted imaging (SWI). DTI studies have demonstrated that even a single MB is associated with widespread alterations in WM integrity (10–12). Some research suggests a threshold effect, indicating that more MBs (e.g., ≥3) may be necessary to observe significant cognitive impairment (13, 14).

MBs are closely linked to deficits in various cognitive functions, including executive functioning, processing speed, and attention. When MBs located in specific brain regions—particularly the frontal and temporal lobes—they have been associated with reduced psychomotor speed and attentional capacity (15, 16). Additionally, MBs in deeper brain structures have been associated with impairments in specific cognitive areas (17–19) with memory being particularly vulnerable in cases involving temporal lobe MBs (20).

The prognostic value of MBs in TBI has been further supported by Aline M. et al., who conducted a systematic review of experimental studies from 2000 to 2014 and concluded that MBs may serve as predictors of outcomes following mild TBI (21). However, region-level analysis by Andreassen et al. found that only the midsagittal region out of the five examined brain regions showed significant co-localization between MBs and DTI abnormalities, suggesting that WM damage extends beyond hemorrhagic lesions (22).

Accurately assessing the extent of brain damage following TBI and predicting patient prognosis–particularly in identifying those who would benefit most from rehabilitation–remains a challenge. While DTI provides valuable insights into the severity of TAI, its routine clinical use is hindered by several technical limitations. These limitations include variability across scanners and protocols, the need for specialized and time consuming post-processing and motion correction. As a result, DTI remains underutilized in routine clinical workflows, despite its proven sensitivity to microstructural damage. Unlike DTI, the assessment of MBs can be easily performed in routine clinical settings. However, their connection to TAI is not yet fully understood. Accurate and automated segmentation of WM structures is essential for robust damage quantification. Comparing available segmentation approaches can help determine the most reliable and clinically applicable methods in TBI.

By clarifying the relationship between MBs and WM microstructure and assessing the effectiveness of tools used to detect such damage, this study aims to contribute to the development of more accessible and reliable neuroimaging biomarkers for cognitive outcomes in TBI.

## 2 Materials and methods

### 2.1 Participants and study design

This study was conducted at the Pécs Diagnostic Centre (PDK) using the Declaration of Helsinki principles. Ethical approval was obtained from the Institutional Review Board of the University of Pécs (No. 4525). Written informed consent was secured from all participants or their legally authorized representatives for the MRI scans used in the study. To ensure complete anonymization, each participant was assigned a unique code name in compliance with the General Data Protection Regulation (GDPR) (23).

The study included 51 participants, divided into three groups: 17 controls subjects (10 women, mean age 55.12 ± 14.47, CON group) and 17 patients with moderate-to-severe TBI who had MBs detected on SWI images (2 women, mean age 54.65 ± 14.14, MBP group), and 17 patients with moderate-to-severe TBI without MBs (6 women, mean age 55.47 ± 14.57, MBN group). TBI severity was classified using the Mayo Classification System for TBI Severity (24). Participants were age-matched (±3 years) into triplets to minimize confounding variables.

Inclusion criteria for the TBI cohorts required the absence of pre-existing neurological or psychiatric conditions and no comorbidities associated with MBs. Participants were excluded if they had contraindications for MRI or if imaging quality was insufficient (25, 26). A summary of the epidemiological data is provided in Table 1.

**Table 1:** Summary table of epidemiological data.

Group characteristics	MBP	MBN	CON
Age (Mean ± SD)	54.65 ± 14.14	55.47 ± 14.57	55.12 ± 14.47
Sex (Male–Female)	15–2	11–6	7–10
MB median number (range)	3 (1–21)	0	0

MBP, Microbleeds Positive; MBN, Microbleeds Negative; CON, Control.

### 2.2 Imaging protocol

MRI examinations were performed using a Siemens MAGNETOM Prisma 3 Tesla scanner (Erlangen, Germany) with a 32-channel head coil and 60 directions (27). The imaging protocol was optimized to detect structural and microstructural changes associated with TBI. The diffusion-weighted imaging protocol included one non-diffusion-weighted (b0) volume and 30 diffusion-weighted volumes with a b-value of 1,000 s/mm^2^ with 2x2x2 mm voxelsize, acquired with both anterior-to-posterior (AP) and posterior-to-anterior (PA) phase encoding directions to enable susceptibility distortion correction. Imaging parameters were consistent throughout the study to ensure reproducibility, with no major protocol modifications or upgrades. Details of the applied sequences and their parameters are summarized in Table 2.

**Table 2:** MRI acquisition parameters used in the study.

Modalities	TR	TE	FoV	Slice thickness
MPRAGE	2,530 ms	3,37 ms	256 mm	1 mm
SWI	27 ms	20 ms	220 mm	1,5 mm
DTI	7,700 ms	68 ms	256 mm	2 mm

MPRAGE, Magnetization prepared rapid acquisition gradient echo; SWI, Susceptibility weighted imaging; DTI, Diffusion tensor imaging; TR, Time of repetition; TE, Time of echo; FoV, Field of view.

### 2.3 Image preprocessing

The raw DICOM images, containing patient metadata and imaging data, were converted to NIFTI format using the “dcm2niix” converter (28). Image preprocessing was performed with the FMRIB Software Library (FSL) version 6.0.7.18, a widely used software for brain imaging analysis developed by the Oxford Centre for Functional MRI of the Brain (FMRIB), University of Oxford (29).

Susceptibility-induced off-resonance fields and undistorted b0 images were estimated with FSL’s *topup* using the first two b0 volumes acquired with opposite phase-encoding directions (30). A binary brain mask for the diffusion data was then generated with FSL’s Brain Extraction Tool (BET) (31), on the average of the two undistorted b0 images. Subsequently, susceptibility- and eddy current–related distortions, as well as subject motion, were corrected, while outlier slices with mean intensities at least three standard deviations lower than expected were identified and replaced through Gaussian Process prediction, using the GPU-accelerated *eddy_cuda11.0* implementation (32) with the resamp = lsr option. These preprocessing steps improved DTI data quality and ensured robust and reliable analyses.

### 2.4 Lesion detection and localization

Two independent authors (D. B. and Z. P.), each with more than 4 years of experience in human brain MRI data processing, evaluated the images individually. Final lesion counts were determined based on consensus. Lesions were validated by A. T., a neuroradiologist with over 10 years of experience. MBs were defined as ovoid or curvilinear hypointensities on SWI.

**Figure 1:**
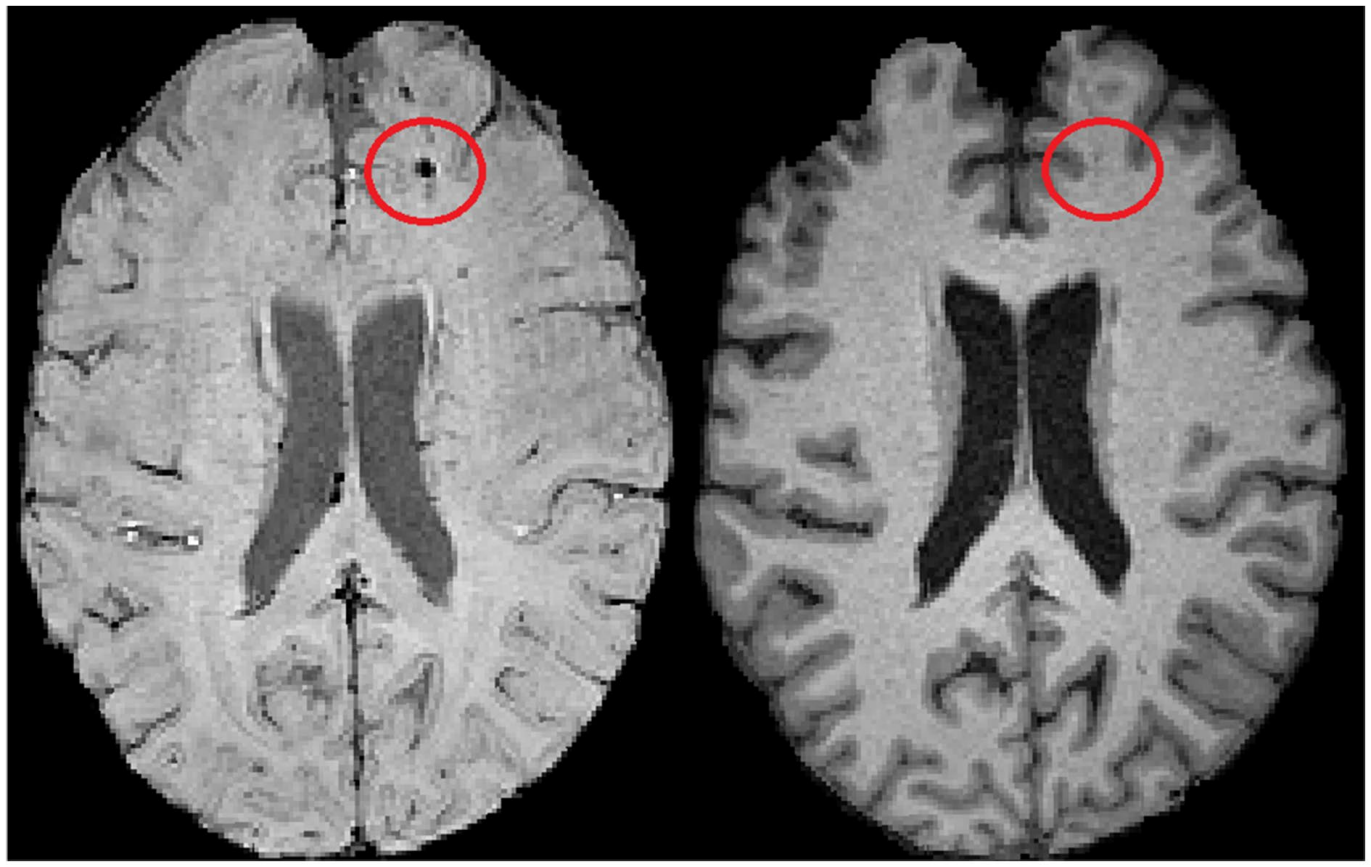
A located MB displayed on SWI (left), with the co-registered MPRAGE image (right).

SWI data were co-registered with high-resolution T1-weighted MPRAGE images using FSL’s FLIRT module to identify MBs precisely (33). This process allowed for precise mapping of lesions in a 3D anatomical space, which was further verified across axial, coronal, and sagittal planes to ensure accurate localization. The identified MB is shown in Figure 1.

### 2.5 White matter masks

To assess WM microstructural alterations, we reconstructed three types of WM masks using complementary methodologies.

### 2.6 Whole white matter reconstruction

Whole WM masks were generated using FreeSurfer version 7.4’s automated recon-all segmentation and parcellation pipeline. This tool processes high-resolution T1-weighted MPRAGE images to perform surface-based and volumetric segmentation, leveraging image intensity data, probabilistic atlases, and topological constraints to delineate cortical and subcortical structures. FreeSurfer offers improved anatomical accuracy, particularly in differentiating adjacent tissue types (34).

After segmentation, the WM volumes of both cerebral hemispheres, corpus callosum (CC) and the brainstem were extracted, combined, and converted into NIFTI format. The resulting volume was then binarized to generate whole WM mask, which was subsequently used to extract global DTI metrics.

### 2.7 Tractography-based white matter reconstruction

We employed probabilistic tractography using FSL’s BEDPOSTX and PROBTRACKX modules to obtain tract-level information. These tools estimate within-voxel fiber orientation distributions and generate probabilistic streamlines across the brain, allowing more accurate mapping of complex fiber architectures, particularly in regions with fiber crossings (35).

Individual tract reconstruction was performed using XTRACT, a fully automated tool within FSL’s Diffusion Toolbox (FDT), which segments 42 major WM tracts based on standard space templates. The output tract masks were registered to each subject’s native space, binarized, and summed to create a comprehensive, subject-specific WM mask derived from tractography. The comprehensive WM was combined with the CC mask from FreeSurfer’s segmentation as the FSL XTRACT tool does not reconstruct the CC. This combined mask reflects the anatomical pathways of WM (36).

### 2.8 Corpus callosum

**Figure 2:**
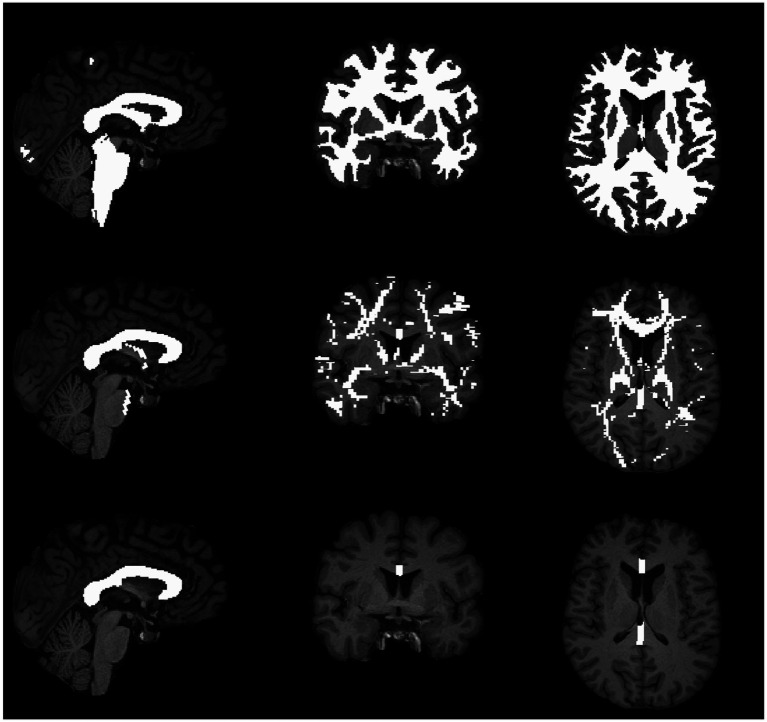
Reconstructed white matter masks overlaid on the MPRAGE anatomical image. The top panel illustrates the global white matter mask, the middle panel shows the whole-brain white matter mask derived from tractography-based reconstruction, and the bottom panel displays the isolated mask of the corpus callosum.

The CC was also analyzed separately due to its highlighted importance in Adams’ classification and its particular vulnerability to shear forces in TAI. Although the brainstem is similarly emphasized in this context, the known variability related to its position within the FOV did not allow for reliable separate analysis. Using FreeSurfer’s segmentation, the five midsagittal CC subdivisions (posterior, mid-posterior, central, mid-anterior, and anterior) were merged into a single volumetric mask. The merged region was converted to NIFTI format and binarized for analysis. The reconstructed WM masks are shown in Figure 2.

### 2.9 Diffusion analysis

For diffusion evaluation, we utilized the FDT modules, which provide tools for performing DTI analysis and obtaining quantitative metrics of WM integrity. Diffusion metric maps for FA and MD were computed using the FDT’s DTIFIT module with the weighted least squares (−wls) option enabled (37). We then projected our binarized WM masks onto the diffusion parameter maps and calculated each mask’s mean of FA, MD. The accuracy of the registrations and the successful execution of FSL BET were confirmed before beginning the evaluation.

### 2.10 Statistics

Statistical analyses were performed using IBM SPSS Statistics (version 29). Data normality was assessed with the Shapiro–Wilk test. Due to the presence of cluster-like triplet data structures, within-subject comparisons were conducted using either repeated measures ANOVA, with Mauchly’s test of sphericity, or the non-parametric Friedman test, depending on the distributional characteristics of the data. *Post hoc* tests were applied in both cases to identify pairwise differences between groups. Given that the familywise Type I error rate for the LSD procedure remains at the nominal alpha level when comparing three groups (38), no correction was necessary for our *post hoc* tests (39). Results were considered statistically significant at *p*-values less than 0.05. According to the results of the Shapiro–Wilk normality tests, repeated-measures ANOVA was applied for FA across all three masks and for MD within the tractography-based WM mask, whereas the Friedman test was used for MD in the whole WM mask and the CC mask.

Generalized linear models (GLiMs) were used to further assess group-level effects and the influence of covariates. For normally distributed dependent variables, a standard GLiM was applied. When normality assumptions were violated, a GLiM with a gamma distribution and log link function was used to determine statistical significance. Parameter estimation in both models was carried out using Maximum Likelihood Estimation (MLE). Age and the number of MBs were included as covariates, while sex was entered as a categorical factor.

### 2.11 Tract-based spatial statistics

We employed Tract-Based Spatial Statistics (TBSS) to perform voxel-wise statistical analyses of WM microstructure across subjects. TBSS uses nonlinear registration to align individual FA maps to a standard space and then reconstructs a mean FA skeleton representing the centers of all major WM tracts common to the group. Each subject’s diffusion parameters were then projected onto this skeleton to enable accurate voxel-wise comparisons while minimizing the influence of partial volume effects and misalignment (40). The age and sex variables were involved into the analysis as covariates.

## 3 Results

A total of 94 MBs were detected on SWI modalities, with a median MB of 3.

In the analysis of whole WM across the three groups pairwise *post hoc* comparisons revealed significantly higher MD values in the MBP group compared to controls (*p* = 0.016). No significant pairwise differences were observed between the MBP and MBN groups or between the MBN group and controls.

**Figure 3:**
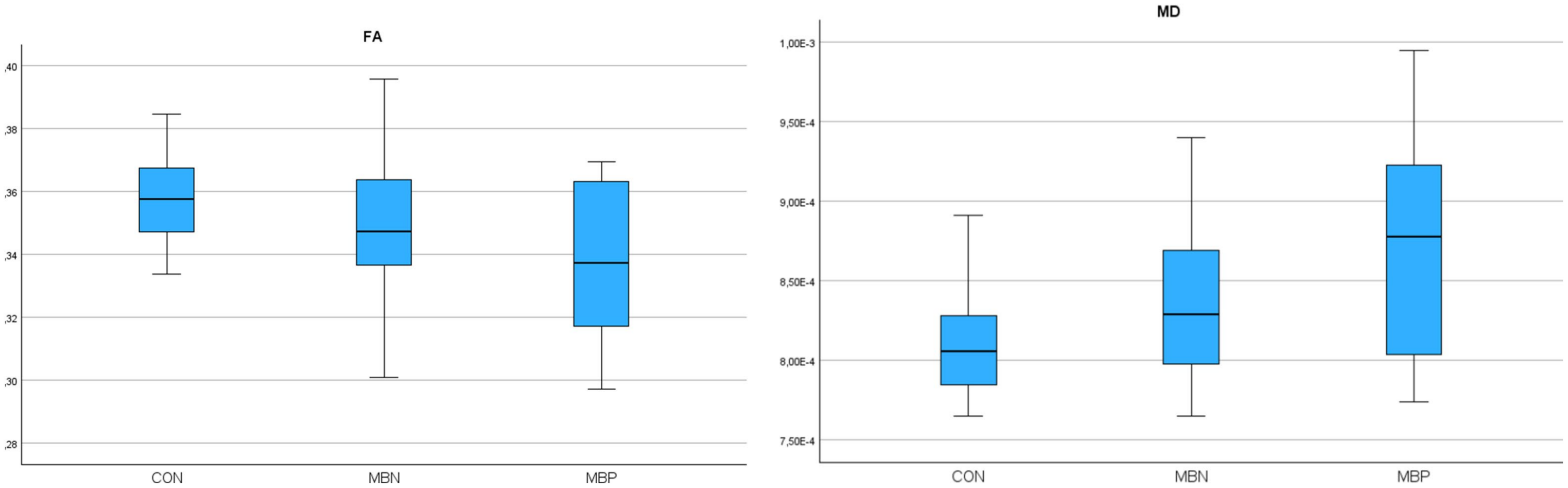
Boxplots illustrating fractional anisotropy (FA), mean diffusivity (MD) parameters across groups for the reconstructed whole white matter.

Based on the results of GLiM analysis, FA values were negatively associated with male sex (*B* = −0.017, *p* = 0.004) and age (*B* = −0.001, *p* = 0.005). For MD, significant positive associations were found with male sex (*B* = 3,598E-5 mm^2^/s, *p* = 0.007) and age (*B* = 2,184E-6 mm^2^/s, *p* < 0.001). The number of MBs showed significant associations with FA decrease (*B* = −0,001, *p* = 0,037) and MD increase (*B* = 3,988E-6 mm^2^/s, *p* = 0,008). A comprehensive presentation of these findings is shown in Table 3. The group-wise distribution of DTI parameters within the reconstructed whole WM is illustrated in Figure 3.

**Table 3:** Group comparisons and GLiM-based analysis of DTI metrics using whole WM masks.

Whole WM mask results *p* values comparison		
DTI parameter	FA	MD
Friedman/Repeated Measure Anova	0.064	0.051
MBN-CON	0.568	0.346
MBP-CON	0.052	**0.016**
MBP-MBN	0.095	0.145

Parameter	B	CI 95%	*p* values
FA			
Sex (male)	−0.017	−0.029 — −0.005	**0.004**
Age	−0.001	−0.001 — −0.0002	**<0.001**
MB number	−0.001	−0.003 — −7.967E-5	**0.037**
MD			
Sex (male)	3.598E-5 mm^2^/s	9.159E−6 — 6.28E-5 mm^2^/s	**0.007**
Age	2.184E-6 mm^2^/s	1.27E-6 — 3.097E-6 mm^2^/s	**<0.001**
MB number	3.988E-6 mm^2^/s	1.01E-6 — 6.966E-6 mm^2^/s	**0.008**

FA, Fractional anisotropy; MD, Mean diffusivity; CI, Confidence interval; B, Unstandardized regression coefficient.

Statistically significant results (*p* < 0.05) are highlighted in bold.

In tractography based DTI analysis, repeated measures ANOVA indicated statistically significant group effects for MD (*p* = 0.011). Pairwise *post hoc* comparisons revealed that the MBP group significantly differed from controls in FA (*p* = 0,042) and in MD (*p* = 0.01). No significant pairwise differences were observed between the MBP and MBN groups or between the MBN group and controls.

**Figure 4:**
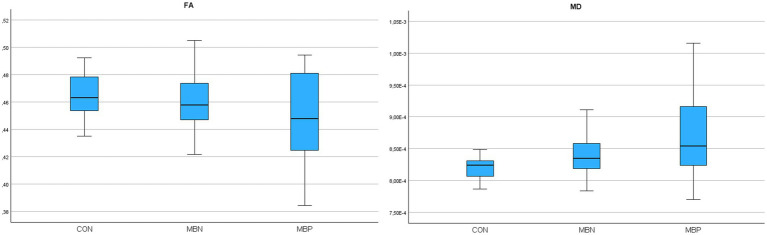
Boxplots illustrating fractional anisotropy (FA), mean diffusivity (MD) parameters across groups for the probabilistically reconstructed tractography based white matter.

GLiMs further supported these findings. FA was negatively associated with male sex (*B* = −0.015, *p* = 0.017), age (*B* = −0.001, *p* < 0.001) and MB number (*B* = −0.001, *p* = 0,039). In the MD model age (*B* = 1,86E-6 mm^2^/s, *p* < 0.001) and MB number (*B* = 3,07E-6 mm^2^/s, *p* = 0.024) were significant predictors. A comprehensive presentation of the findings is shown in Table 4. The group-wise distribution of DTI parameters within the probabilistically reconstructed tractography based WM is illustrated in Figure 4.

**Table 4:** Group comparisons and GLiM-based covariate analysis of DTI metrics in the probabilistically reconstructed tractography based white matter.

Tractography based mask results *p* values comparison		
DTI Parameter	FA	MD
Repeated measures Anova	0.055	**0.011**
MBN-CON	0.582	0.113
MBP-CON	**0.042**	**0.01**
MBP-MBN	0.098	0.097

Parameter	B	CI 95%	*p* values
FA			
Sex (male)	−0.015	−0.027 — − 0.003	**0.017**
Age	−0.001	−0.0014 — − 0.0006	**<0.001**
MB number	−0.001	−0.003 — −6.83E-5	**0.039**
MD			
Sex (male)	2.33E-5 mm^2^/s	−6.31E-7 — 4.72E-5 mm^2^/s	0.056
Age	1.86E-6 mm^2^/s	1.04E-6 — 2.67E-6 mm^2^/s	**<0.001**
MB number	3.07E-6 mm^2^/s	4.1E-7 — 5.72E-6 mm^2^/s	**0.024**

FA, Fractional anisotropy; MD, Mean diffusivity; CI, Confidence interval; B, Unstandardized regression coefficient.

Statistically significant results (*p* < 0.05) are highlighted in bold.

In the case of the CC repeated measures ANOVA indicated statistically significant differences for FA (*p* = 0.005) and non-parametric Friedman tests indicated significant differences in MD (*p* = 0.014). The MBP group showed in *post hoc* pairwise comparisons significantly altered values compared to controls in FA (*p* = 0.008) and MD (*p* = 0.004). FA values differed significantly between the MBP and MBN groups (*p* = 0.007).

**Figure 5:**
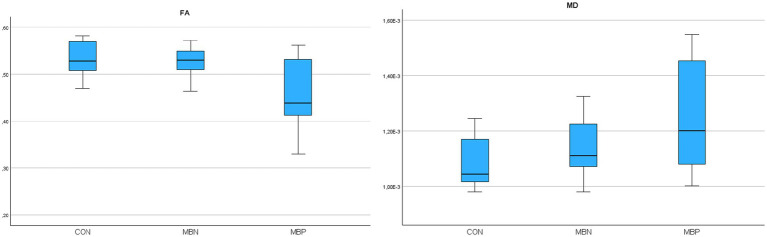
Boxplots illustrating fractional anisotropy (FA), mean diffusivity (MD) parameters across groups for the reconstructed corpus callosum.

In the GLiM, several significant associations were observed. The FA model showed a significant negative association with male sex (*B* = −0.046 mm^2^/s, *p* = 0.007), age (*B* = −0,002, *p* = 0.006) and the number of MBs (*B* = −0.007, *p* < 0.001). In the MD model, significant positive associations were observed with male sex (*B* = 0.0001 mm^2^/s, *p* = 0.004), age (*B* = 5,35E-6 mm^2^/s, *p* < 0.001) and MB number (*B* = 9,68E-6 mm^2^/s, *p* = 0.015). A comprehensive presentation of the findings is shown in Table 5. The group-wise distribution of DTI parameters within the reconstructed CC is illustrated in Figure 5.

**Table 5:** Group comparisons and GLiM-based covariate analysis of DTI metrics in the corpus callosum.

Corpus callosum mask results *p* values comparison		
DTI Parameter	FA	MD
Friedman/ Repeated measures Anova	**0.005**	**0.014**
MBN-CON	0.851	0.086
MBP-CON	**0.008**	**0.004**
MBP-MBN	**0.007**	0.23

Parameter	B	CI 95%	*p* values
FA			
Sex (male)	−0.046	−0.08 — −0.012	**0.007**
Age	−0.002	−0.003 — −0.0005	**0.006**
MB number	−0.007	−0.01 — −0.003	**<0.001**
MD			
Sex (male)	0.0001 mm^2^/s	3.315E-5 — 0.00017 mm^2^/s	**0.004**
Age	5.35E-6 mm^2^/s	2.96E-6 — 7.74E-6 mm^2^/s	**<0.001**
MB number	9.68E-6 mm^2^/s	1.884E-6 — 1.748E-5 mm^2^/s	**0.015**

FA, Fractional anisotropy; MD, Mean diffusivity; CI, Confidence interval; B, Unstandardized regression coefficient.

Statistically significant results (*p* < 0.05) are highlighted in bold.

**Figure 6:**
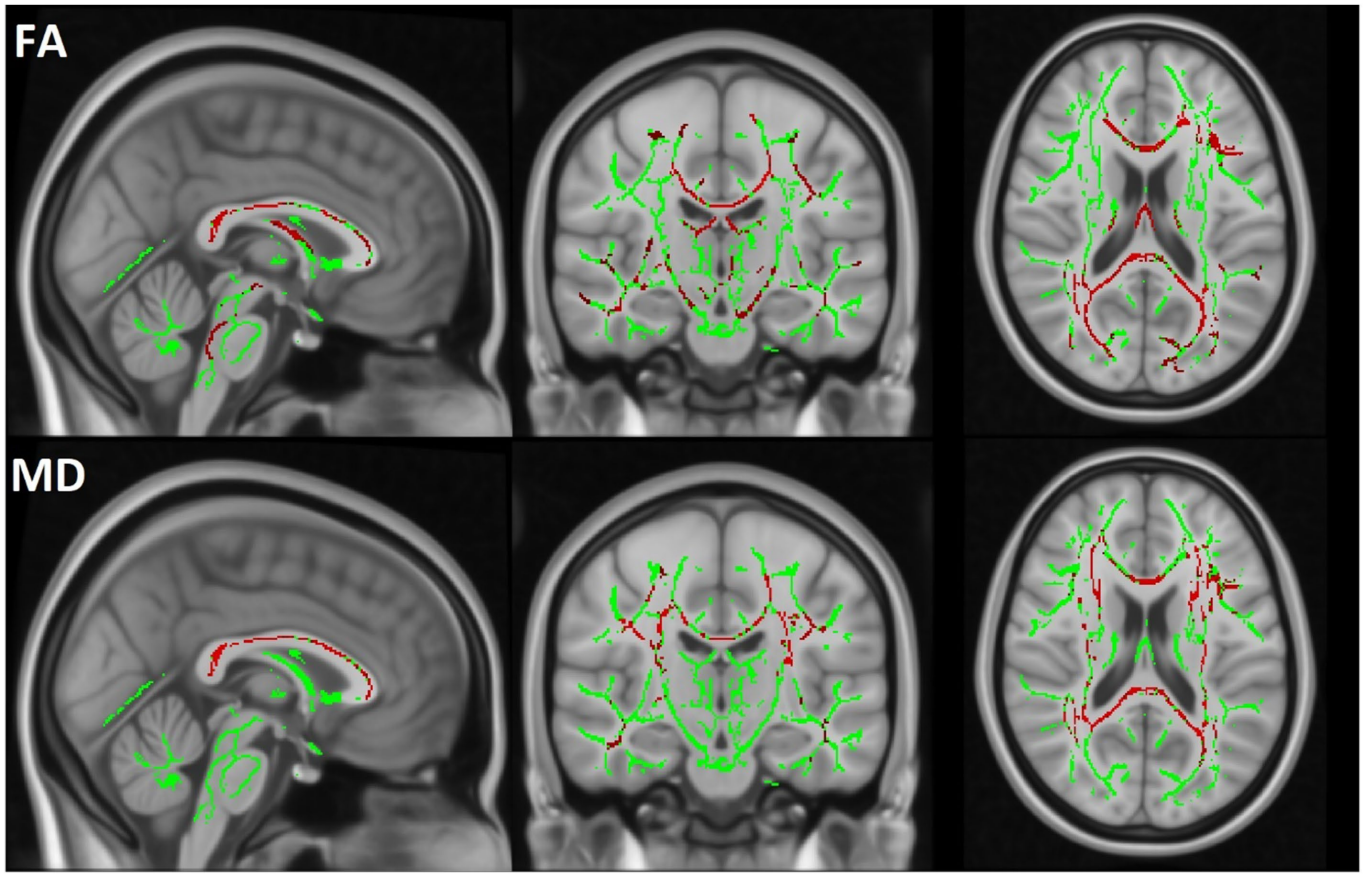
The TBSS results comparing the MBP and CON groups are displayed. The skeleton mask is overlaid in green on the MN152 template, while voxels exceeding the 0.95 threshold are highlighted in red along the skeleton. The first row represents FA and the second row MD results.

**Figure 7:**
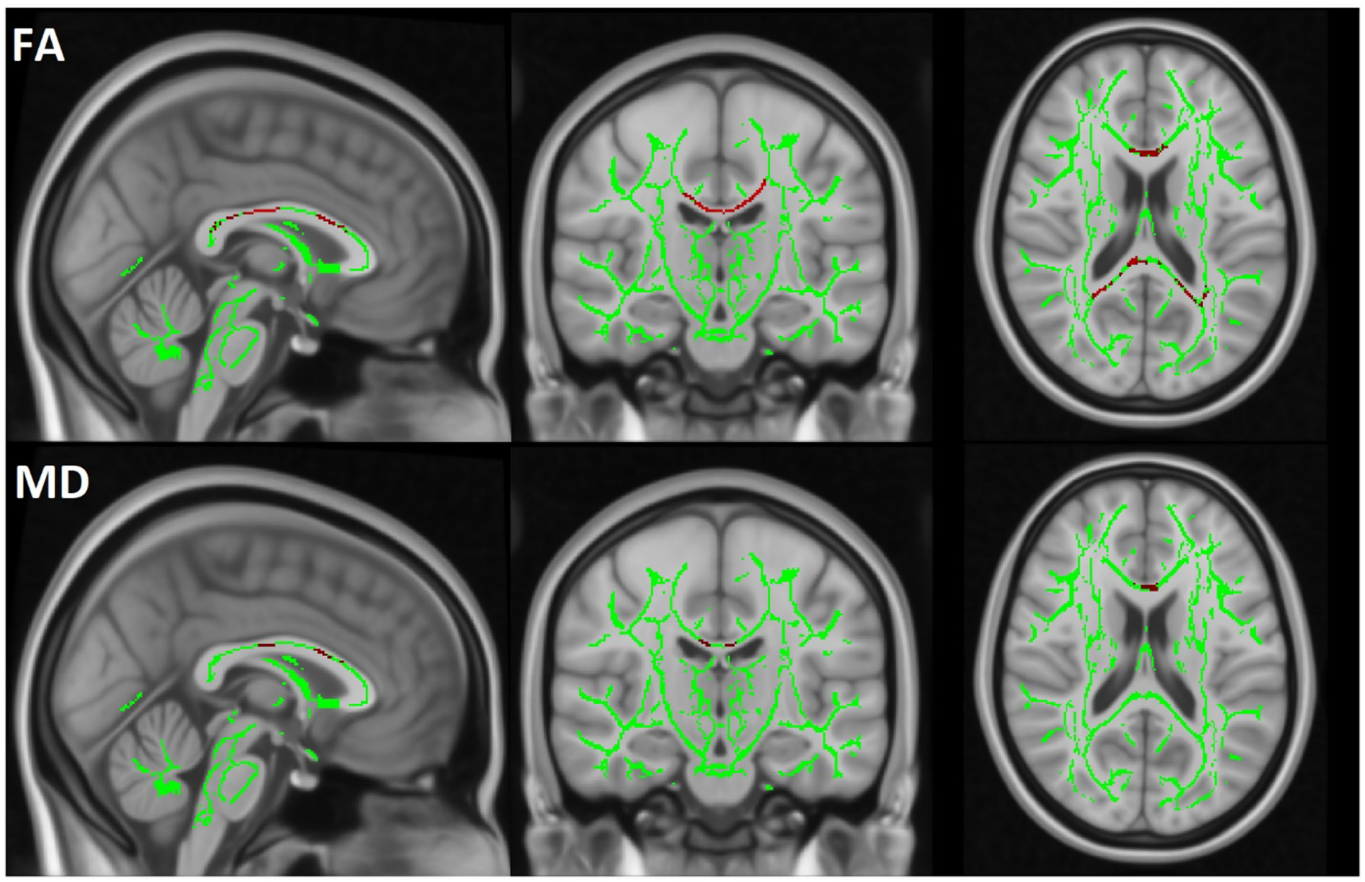
The TBSS results are displayed by comparing the MBP and MBN groups. The skeleton mask is overlaid in green on the MN152 template, while voxels exceeding the 0.95 threshold are highlighted in red along the skeleton. The first row represents FA and the second row MD results.

TBSS analysis revealed a significant decrease in FA and an increase in MD in the MBP group compared to the control group. Additionally, a significant decrease in FA and an increase in MD were observed when comparing the MBP group to the MBN group. The statistical results of the TBSS analysis are presented in Table 6. The significant results of the MBP-CON group comparison are shown in Figure 6 while the significant results of the MBP vs. MBN group comparison are displayed in Figure 7.

**Table 6:** The table presents the highest 1-*p* values obtained from the TBSS analysis.

DTI Parameters	MBP – MBN	MBN - CON	MBP - CON
FA	0.508 ↑ **0.988 ↓**	0.339 ↑ 0.563 **↓**	0.102 ↑ **0.995 ↓**
MD	**0.957** ↑ 0.189**↓**	0.85 ↑ 0.301 **↓**	**0.992** ↑ 0.071 **↓**

Statistically significant results (*p* < 0.05) are highlighted in bold.

## 4 Discussion

In this retrospective study, we analyzed a cohort of patients who underwent evaluation using an MRI protocol optimized for TBI assessment. Our primary aim was to explore the clinical relevance of MBs in relation to overall WM integrity. Furthermore, we examined the sensitivity of different automated segmentation approaches in detecting TAI, aiming to assess their efficacy in identifying subtle microstructural alterations.

To ensure robust assessment of WM alterations, we applied multiple complementary analytic approaches, including region-based segmentation and voxel-wise statistics. Covariates such as age and sex were also accounted for, given their known influence on WM microstructure. The consistency of findings across different WM analysis approaches—particularly in the CC—highlights the robust relationship between MBs and disrupted WM integrity. Notably, the observed decrease in FA and increases in MD in MBP patients suggest widespread microstructural damage beyond MBs. Importantly, the observed alterations agree with a comprehensive systematic review by Wortzel et al., which synthesized results from 66 studies examining diffusion changes following acute and subacute TBI. Their analysis identified decreased FA alongside increased MD and RD as the most consistent diffusion alterations associated with TBI in adults (41). The direction and pattern of DTI parameter changes in our cohort align with this broader literature. The TBSS analysis further reinforced the relevance of MBs, as significant WM alterations were observed in the MBP group compared to CON group in FA and MD, whereas the MBN group showed no significant alteration. In the comparison between the MBP and MBN groups, FA was significantly reduced, while MD were increased, further supporting the presence of more severe injury in the MBP group.

Probabilistic tractography-based analyses demonstrated greater sensitivity, identifying significant associations that were not detectable using whole WM segmentation both in the pre-hoc and pairwise comparisons. Across all three white matter masks, both FA and MD showed significant associations with the number of MBs. This aligns with prior findings suggesting that not only the presence but also the number and distribution of MBs may be linked to clinical outcomes such as post-TBI depression (42) and poorer cognitive performance, including reduced short-term memory (43). The broader impact of MBs beyond their focal location is further supported by the study of Haber et al., who found limited topographical overlap between cerebrovascular reactivity (CVR) abnormalities and diffusion alterations (44). This spatial dissociation between vascular and axonal injury highlights TBI pathology’s complex and widespread nature. Similarly to our study, Juho Dahl and colleagues reported worse global WM integrity, reflected by lower FA and higher MD, in MB-positive TBI patients compared to MB-negative patients. However, after adjusting for clinical severity indicators such as the Glasgow Coma Scale and post-traumatic amnesia duration, these differences disappeared (12). Still, in contrast, another study found a significant association between the presence of MBs and Glasgow Coma Scale scores (45). This finding suggests that MBs may be more prevalent in patients with more severe injuries, yet their absence does not exclude the potential for poor outcomes. In their TBSS analysis, Dahl et al. identified significant differences in FA between MB-negative patients and controls, contrasting our results. This discrepancy may be explained by their substantially larger sample size.

The FA differences identified in the TBSS comparison between MBP and MBN patients were primarily localized to the midsagittal region. This aligns with the mask-level results where the CC showed significant FA alteration between the MBP and MBN group. Importantly, in our cohort, the number of MBs significantly influenced the DTI parameters in the CC, reinforcing the link between MBs burden and WM disruption. This central vulnerability is supported by prior region-level analyses by Andreassen et al. (22) They found that among five examined brain regions, only the midsagittal area exhibited significant co-localization between MBs and diffusion abnormalities, highlighting the susceptibility of central WM structures to trauma-related microstructural damage. Similarly, Tóth et al. reported that lesions affecting central brain regions were associated with poorer clinical outcomes in MB-positive patients (46). Our findings also parallel those of Moen et al., who used manually defined ROIs to observe significantly reduced FA values in MB-positive TBI patients relative to both MB-negative patients and healthy controls. The most pronounced differences were again localized to the corpus callosum, further supporting its sensitivity to TAI and its relevance as a core region for detecting MB-related microstructural changes (47).

These patterns collectively suggest that the presence and the number of MBs may serve as markers of TAI. Moreover, age and male sex consistently emerged as independent contributors to WM deterioration, aligning with existing evidence on demographic vulnerability in TAI. These factors were significantly related to DTI metrics independent of MB status. The influence of age on DTI parameters is consistent with prior findings reported in both mild (48–50) and moderate-to-severe traumatic brain injury populations (51). There is extensive literature on the role of age in traumatic brain injury, supporting its association with later cognitive and functional decline. However, the role of sex in TBI outcomes remains complex and inconsistent across the literature. Some studies have reported that male patients show greater WM microstructural damage and longer symptom duration, as indicated by decreased FA in key tracts such as the uncinate fasciculus (52). In contrast, other research suggests that female sex is associated with poorer post-concussive symptom outcomes, particularly during the childbearing years, potentially linked to hormonal influences affecting recovery processes (53). These divergent findings highlight the multifactorial and heterogeneous nature of sex-related vulnerability in TBI, shaped by biological, hormonal, and psychosocial factors.

Despite the strengths of our study, several limitations must be acknowledged. While our sample size was sufficient to detect group-level differences, it may limit the generalizability of our findings. Another limitation concerns the uncertain origin of the MBs detected in our cohort. Although all patients sustained TBI, not all MBs observed on SWI can be confidently attributed to trauma. MBs are more prevalent in older individuals and may also result from non-traumatic causes. Since the underlying cause of each MB could not be established in this retrospective study, it is possible that a portion of the detected lesions were incidental or unrelated to the trauma itself. In the case of the whole WM mask analysis, the extent of brainstem coverage may vary between subjects, as individual positioning within the scanner affects how much of the brainstem falls within the FoV. However, the observed differences in this analysis were consistent with the findings obtained from the other analytical approaches, supporting the validity and interpretability of the results derived from the whole WM mask. Future research should aim to replicate these findings in larger cohorts, ideally with longitudinal follow-up to assess the progression of microstructural changes over time.

## 5 Conclusion

Our findings highlight MBs as potential markers of more extensive WM injury in moderate-to-severe TBI. The increase in MBs suggests even greater WM damage, indicating a progression of microstructural alterations. On a global scale, tractography enhances the sensitivity in detecting structural alterations compared to traditional segmentation techniques. Examining central WM areas holds significant importance in uncovering the relevance of microbleeds. Our results may therefore have clinical implications, suggesting that patients with MBs could benefit from more intensive monitoring and tailored rehabilitation strategies.

## Data Availability

The raw data supporting the conclusions of this article will be made available by the authors, without undue reservation.
